# Gender Differences in X (Formerly Twitter) Use Among Oncology Physicians at National Cancer Institute–Designated Cancer Centers: Cross-Sectional Study

**DOI:** 10.2196/66054

**Published:** 2025-03-11

**Authors:** Vivian Tieu, Sungjin Kim, Minji Seok, Leslie Ballas, Mitchell Kamrava, Katelyn M Atkins

**Affiliations:** 1 California University of Science and Medicine Colton, CA United States; 2 Department of Radiation Oncology Cedars-Sinai Medical Center Los Angeles, CA United States

**Keywords:** social media, gender disparities, gender differences, cross-sectional study, twitter, oncology

## Abstract

This cross-sectional study evaluated gender parity in the oncology workforce on social media, demonstrating that women oncologists are enriched on X, with higher self-engagement, suggestive of a heightened motivation for professional X use.

## Introduction

In 2023-2024, women comprised 55% of matriculating US medical students but continue to be underrepresented in academic medicine [1,2]. This is particularly true in the workforce and leadership of oncology disciplines [2]. Social media platforms have gained popularity for professional development [3], though data characterizing gender parity in the social media oncology workforce are lacking and studied herein.

## Methods

### Overview

Twenty regionally distinct National Cancer Institute (NCI)–designated cancer center websites were accessed from December 2023 to July 2024 (Table 1) and physician demographic information was collected. Apparent gender (binary) was assigned by a single researcher (VT) using names, pronouns, and/or public profile images. Among physicians on X, publicly available data were manually collected. Physicians with missing numbers of “likes” (public reporting discontinued June 12, 2024) were excluded. Statistical analysis by X use and gender was performed using Wilcoxon rank sum and chi-square (or Fisher exact) tests for continuous and categorical variables, respectively. Analyses were performed using R (version 4.0.5; The R Foundation) with 2-sided tests with *P*≤.05.

**Table 1:** National Cancer Institute–designated cancer centers included in this study.

Institution name	City	State	Physicians (total; n=2908), n (%)	Physicians on X (n=1068), n (%)
O’Neal Comprehensive Cancer Center	Birmingham	AL	45 (1.5)	29 (2.7)
University of Arizona Cancer Center	Tucson	AZ	54 (1.9)	23 (2.2)
Mayo Clinic Cancer Center	Phoenix, Jacksonville, and Rochester	AZ, FL, and MN	156 (5.4)	70 (6.6)
Chao Family Comprehensive Cancer Center	Orange	CA	126 (4.3)	36 (3.4)
University of Colorado Cancer Center	Aurora	CO	147 (5.1)	51 (4.8)
Yale Cancer Center	New Haven	CT	353 (12.1)	148 (13.9)
Sylvester Comprehensive Cancer Center	Miami	FL	149 (5.1)	71 (6.6)
Winship Cancer Institute	Atlanta	GA	262 (9.0)	106 (9.9)
University of Hawaii Cancer Center	Honolulu	HI	28 (1.0)	4 (0.4)
Robert H. Lurie Comprehensive Cancer Center	Chicago	IL	203 (7.0)	86 (8.1)
Indiana University Melvin and Bren Simon Comprehensive Cancer Center	Indianapolis	IN	148 (5.1)	56 (5.2)
Holden Comprehensive Cancer Center	Iowa City	IA	87 (3.0)	24 (2.2)
The University of Kansas Cancer Center	Kansas City	KS	109 (3.7)	29 (2.7)
Markey Cancer Center	Lexington	KY	62 (2.1)	17 (1.6)
Sidney Kimmel Comprehensive Cancer Center	Baltimore	MD	314 (10.8)	110 (10.3)
The Barbara Ann Karmanos Cancer Institute	Detroit	MI	81 (2.8)	25 (2.3)
Alvin J. Siteman Cancer Center	St. Louis	MO	315 (10.8)	118 (11.0)
Dartmouth Cancer Center	Lebanon	NH	153 (5.3)	24 (2.2)
Rutgers Cancer Institute of New Jersey	New Brunswick	NJ	116 (4.0)	41 (3.8)

### Ethical Considerations

This cross-sectional study used publicly available data and was therefore exempt from ethical approval per the Cedars-Sinai Medical Center institutional review board (STUDY00003292). STROBE (Strengthening the Reporting of Observational Studies in Epidemiology) reporting guidelines were followed (Multimedia Appendix 1).

## Results

In total, 2908 physicians’ profiles were analyzed, of which 37% (n=1068) were on X (Table 2). There was a greater proportion of women (vs men) physicians on X in the Northeast (35.1% vs 27.1%) but a smaller proportion in the Midwest (32.2% vs 39.6%; *P*=.03; Table 2). X users accounted for a higher proportion of women (39% vs 35%; *P*=.05), were more likely to hold leadership titles (*P*<.001), and had an advanced dual degree (33% vs 25%; *P*<.001) than non–X users. Among those on X, women (vs men) were less likely to have “professor” status (25% vs 41%; *P*<.001) and leadership titles (*P*=.006) but more likely to have a master’s in public health (9% vs 5%; *P*=.03).

**Table 2:** Characteristics stratified by apparent gender on X.

Variable				Men (n=654)		Women (n=413)		*P* value^a^		
**Region**										.03
	Northeast			177 (27.1)		145 (35.1)				
	Midwest			259 (39.6)		133 (32.2)				
	South			143 (21.9)		88 (21.3)				
	West			75 (11.5)		47 (11.4)				
**Faculty type**										<.001
	None or unknown			37 (5.7)		18 (4.4)				
	Instructor or clinician			0 (0)		1 (0.2)				
	Assistant			173 (26.5)		172 (41.7)				
	Associate			177 (27.1)		117 (28.3)				
	Professor			267 (40.8)		105 (25.4)				
**Number of titles including chair, director, or codirector**										.006
	0			324 (49.5)		247 (60.0)				
	1			168 (25.7)		89 (21.6)				
	2			94 (14.4)		50 (12.1)				
	3+			68 (10.4)		26 (6.3)				
**Subspecialty**										<.001
	Medical oncology			231 (35.4)		166 (40.2)				
	Radiation oncology			53 (8.1)		38 (9.2)				
	Gyn oncology			12 (1.8)		18 (4.4)				
	Surgical oncology			44 (6.7)		46 (11.1)				
	Other			313 (47.9)		145 (35.1)				
**Dual degree**										
	**PhD**								.05	
		No	536 (82.0)		357 (86.4)					
		Yes	118 (18.0)		56 (13.6)					
	**MS**								.92	
		No	575 (87.9)		364 (88.1)					
		Yes	79 (12.1)		49 (11.9)					
	**Master of public health**								.03	
		No	620 (94.8)		378 (91.5)					
		Yes	34 (5.2)		35 (8.5)					
	**Any advanced dual degree**								.86	
		No	442 (67.6)		277 (67.1)					
		Yes	212 (32.4)		136 (32.9)					
Length of training since graduation from medical school (years), median (IQR)				7 (6-9)		7 (6-8)		.02		
**X use variables unadjusted for time**										
	Number of followers, median (IQR)			389.5 (146-1119)		305 (112-863)		.002		
	Number of accounts followed by the physicians, median (IQR)			223 (80-521)		219 (82-478)		.78		
	Total number to tweets, median (IQR)			168.5 (38-675)		131 (22-514)		.02		
	Total number of media posts, median (IQR)			15 (2-67)		9 (2-47)		.09		
	Number of liked posts, median (IQR)			352 (35.5-1683)		441 (52-1697.5)		.23		
**X use variables adjusted for time**										
	Time on X (years), median (IQR)			8.1 (5.3-11.6)		6.7 (4.5-10.3)		<.001		
	Average number of followers per year on X, median (IQR)			55.3 (19.4-159.5)		48 (19.6-120.6)		.12		
	Average number of accounts followed per year on X, median (IQR)			29.7 (10.7-71.4)		34 (15.2-73.0)		.09		
	Average number to tweets per year on X, median (IQR)			22.0 (5.2-91.3)		21.4 (4.6-72.9)		.30		
	Average number of media posts per year on X, median (IQR)			1.9 (0.3-8.6)		1.6 (0.3-7.8)		.54		
	Average number of liked posts per year on X, median (IQR)			45.9 (5.6-235.8)		76.6 (9.7-260.4)		.02		
**Thematic content of X biography**										
	**Job roles**								.82	
		No mention	111 (17.1)		68 (16.5)					
		Mention	540 (83.0)		344 (83.5)					
	**Specialty**								.70	
		No mention	128 (19.7)		77 (18.7)					
		Mention	523 (80.3)		335 (81.3)					
	**Being a parent**								.002	
		No mention	583 (89.6)		342 (83.0)					
		Mention	68 (10.5)		70 (17.0)					
	**Spouse**								.25	
		No mention	598 (91.9)		370 (89.8)					
		Mention	53 (8.1)		42 (10.2)					
	**Institution**								.98	
		No mention	209 (32.1)		132 (32.0)					
		Mention	442 (67.9)		280 (68.0)					
	**Personal interests (eg, hobbies and activities)**								.09	
		No mention	565 (86.8)		342 (83.0)					
		Mention	86 (13.2)		70 (17.0)					

^a^*P* values were determined using the Wilcoxon rank sum test for continuous variables and chi-square or Fisher exact test for categorical variables, where appropriate.

Overall, women (vs men) had significantly fewer followers (mean 305 vs 390, *P*=.002) and tweets (mean 131 vs 169, *P*=.02). Adjusting for fewer years on X, women showed similar influence as men (mean 48 vs 55 followers per X-year, *P*=.12) but a higher rate of liking posts (mean 77 vs 46 per X-year, *P*=.02). Women (vs men) were more likely to mention being a parent in their biography (17% vs 10%, *P*=.002), but no differences were noted in other content variables (*P*>.05).

## Discussion

### Principal Results

In this cross-sectional study evaluating gender parity in the oncology workforce on social media, we observed women physicians on X being less likely to hold professor status and leadership titles. As seen on X, particularly in male-dominated fields of radiation and surgical oncology, the proportion of women was significantly higher than published workforce estimates (radiation oncology: 42% vs 31%, *P*=.04; surgical oncology: 51% vs 39%, *P*=.03) [2]. These data are suggestive of motivational and/or behavioral differences in X use by gender.

### Comparisons With Prior Work

We observed that women were more likely to mention being a parent, consistent with studies describing higher engagement in fostering support and community [3]. Further, women “liked” more posts, which perhaps parallels the expected levels of “friendliness” and tone softening in women’s professional communications, which has been associated with increased emotional labor. Indeed, content language analyses have demonstrated that women use exclamation points more frequently than men and as markers of “friendly interaction” [4]. Behavioral psychology studies report that women have higher engagement in emotional labor practices, which may drain resources without equitable rewards, contributing toward the underrepresentation of women in leadership positions [5]. These findings support continued evaluation of motivational and/or behavioral differences in professional social media use.

Men physicians are more likely to hold “verified” X accounts (verification is thought to add a degree of validity) [6] and report professional benefits from social media use, such as invited talks [7], consistent with studies reporting that women X users face challenges in popularity and influence at academic meetings despite comparable activity [8]. Thus, while social media offers a platform for connection and visibility [9], these findings underscore the need for ensuring equitable opportunities moving forward.

### Limitations

Institutional websites may be inaccurate, incomplete, and/or outdated. Gender classification may be inaccurate and impart classification bias. Publicly available X data are more limited than prior studies [10]. Verified status was not analyzed due to low occurrence (0.2% of accounts).

### Conclusions

Women oncologists are enriched on X, with higher self-engagement, suggestive of a heightened motivation for professional X use. Future longitudinal studies examining the role of emotional labor and network support in motives for social media use are warranted.

## Appendix

Multimedia Appendix 1 STROBE guidelines.

## Abbreviations

NCI: National Cancer Institute
STROBE: Strengthening the Reporting of Observational Studies in Epidemiology

## References

[1] 2024 FACTS: Applicants and Matriculants Data. AAMC.

[2] Chowdhary M, Chowdhary A, Royce TJ, Patel KR, Chhabra AM, Jain S, Knoll MA, Vapiwala N, Pro B, Marwaha G (2020). Women's representation in leadership positions in academic medical oncology, radiation oncology, and surgical oncology programs. JAMA Netw Open.

[3] Ibrahim H, Anglade P, Abdel-Razig S (2020). The use of social media by female physicians in an international setting: a mixed methods study of a group WhatsApp chat. Womens Health Rep (New Rochelle).

[4] Waseleski C (2006). Gender and the use of exclamation points in computer-mediated communication: an analysis of exclamations posted to two electronic discussion lists. J Comp Mediated Comm.

[5] Vial AC, Cowgill CM (2022). Heavier lies her crown: gendered patterns of leader emotional labor and their downstream effects. Front Psychol.

[6] Rupert D, Shah K, Chen BY, O'Glasser Avital Y, Schiml M, Jain S, Chino F (2022). Sex and location differences in verification status of physician-held social media platform accounts. JAMA Netw Open.

[7] Woitowich NC, Arora VM, Pendergrast T, Gottlieb M, Trueger NS, Jain S (2021). Gender differences in physician use of social media for professional advancement. JAMA Netw Open.

[8] Berger T, Payan N, Fleury E, Davey A, Bryce-Atkinson A, Vasquez Osorio E, Yang Z, McMullan T, Shelley LE, Gasnier A, Bertholet J, Aznar MC, Nailon WH (2022). Gender-related and geographic trends in interactions between radiotherapy professionals on Twitter. Phys Imaging Radiat Oncol.

[9] Lewis JD, Fane KE, Ingraham AM, Khan A, Mills AM, Pitt SC, Ramo D, Wu RI, Pollart SM (2018). Expanding opportunities for professional development: utilization of Twitter by early career women in academic medicine and science. JMIR Med Educ.

[10] Zhu JM, Pelullo AP, Hassan S, Siderowf L, Merchant RM, Werner RM (2019). Gender differences in Twitter use and influence among health policy and health services researchers. JAMA Intern Med.

